# Healthcare workers’ attitudes toward influenza vaccine prescriptions in China

**DOI:** 10.1186/s41256-025-00430-0

**Published:** 2025-08-04

**Authors:** Yanlin Cao, Qing Wang, Jiemi Zhao, Yuyuan Zhang, Ran Huo, Quanle Li, Weizhong Yang, Heya Yi, Luzhao Feng

**Affiliations:** 1 School of Population Medicine and Public Health, Chinese Academy of Medical Sciences & Peking Union Medical College, Key Laboratory of Pathogen Infection Prevention and Control (Peking Union Medical College), Ministry of Education; State Key Laboratory of Respiratory Health and Multimorbidity; Public Health Emergency Management Innovation Center, Beijing, China; 2 Department of International Affairs, Chinese Preventive Medicine Association, Beijing, China; 3 Yale School of Public Health, Yale University, New Haven, United States

**Keywords:** Healthcare workers, Influenza vaccine, Vaccine hesitancy, Vaccination promotion

## Abstract

**Background:**

Vaccine prescription is being implemented and applied in China to bolster promote vaccination campaigns and mitigate vaccine hesitancy. This study aims to investigate the current vaccine recommendation practices among healthcare workers (HCWs) in China and identify the determinants that influence their willingness to provide vaccination prescription, informing interventions to support the implementation of willingness into practice.

**Methods:**

A cross-sectional survey was conducted among HCWs nationwide in China from July 3 to July 10, 2024. The survey questionnaire was distributed via a link provided by an expert-listening platform at the 2024 World Influenza Conference, representing a diverse group across different healthcare institutions. The study used descriptive and logistic regression analyses performed on attitudes toward providing influenza vaccination prescriptions.

**Results:**

Among 3140 responding HCWs, 68.8% of hospital-based HCWs (N = 778/1131) and 61.9% of community-based HCWs (N = 1243/2009) demonstrate a willingness to provide vaccine prescriptions. HCWs with a history of influenza vaccination (adjusted odds ratio [aOR] = 0.30, 95% confidence interval [CI]: 0.23–0.39, *P* < 0.001) were significantly more inclined to provide vaccine prescriptions. Incentives including bonus rewards (aOR = 1.84, 95% CI: 1.40–2.43, *P* < 0.001), and integration into annual/monthly performance evaluations (aOR = 1.60, 95% CI: 1.20–2.13, *P* = 0.001) further enhanced willingness to provide prescriptions. In terms of communication methods, 63.4% of HCWs (N = 1991) identified official public account promotions on WeChat as the most effective for raising vaccination awareness, significantly surpassing provide vaccine prescriptions (8.7%, N = 350).

**Conclusions:**

Our study emphasizes the necessity for further evaluations of vaccine prescription policies to improve the implementation among HCWs. The findings advocate for tailored strategies, including enhanced incentive mechanisms in hospital settings and optimized digital engagement in community health centers, to facilitate effective vaccine prescription practices.

**Supplementary Information:**

The online version contains supplementary material available at 10.1186/s41256-025-00430-0.

## Background

Influenza continues to pose a substantial public health challenge worldwide, resulting in 3–5 million severe cases and 290,000–650,000 deaths annually from respiratory infections. In China, influenza-associated respiratory diseases are responsible for an average of 88,000 excess deaths [1]. Vaccination stands out as one of the most effective and cost-efficient public health interventions for mitigating the burden of infectious diseases [2]. Studies show that influenza vaccinations avert millions of cases and lower the risk of mortality by 31% [3, 4]. However, despite its proven benefits, vaccination uptake is still not at its optimal level. Existing literature indicates that China’s influenza vaccination coverage has remained at 2–4% across the general population from 2010 to 2023 [5]. This highlights the critical need to develop innovative, evidence-based strategies to enhance influenza vaccination rates.

In China, influenza vaccines are primarily recommended for high-risk groups such as the elderly, patients with chronic diseases, and young children [6]. These groups are the main recipients of influenza vaccine prescriptions and targeted interventions. HCWs’ recommendation behaviors contribute to higher influenza vaccination rates. Their impact is mainly reflected in providing vaccine-related information and improving access to vaccination services. In terms of information delivery, a study in the United States found that using electronic health information systems to support vaccine recommendations increased adult influenza vaccination rates to 32%, compared to 26% in the control group [7]. Health education focused on influenza complications has also been effective for high-risk groups. For example, vaccination coverage among patients increased by 3.1% after recommendation interventions [8]. In terms of access, convenient vaccination services are important to increase uptake. Evidence shows that offering vaccinations during healthcare visits is an effective strategy. One U.S. study reported a 4.3% increase in influenza vaccination rates among children when services were provided at the point of care [9].

Globally, many regions demonstrating suboptimal immunization coverage, only Portugal, the United Kingdom, Denmark, and Ireland—have achieved the World Health Organization's (WHO) influenza vaccination coverage target of 75%. Among the 31 countries surveyed, 13 reported vaccination rates below 40% between 2019 and 2023 [10]. Besides, diverse strategies have emerged to enhance vaccination programs. Australia’s pharmacy-based vaccination services demonstrate improved accessibility [11], while India's community HCWs model shows adaptability to resource-limited settings [12]. These examples highlight the importance of context-specific strategies in expanding vaccination coverage.

In China, the overall influenza vaccination coverage vaccination rate was 3.2% in 2020–2021 and 2.5% in 2021–2022 [13]. The National Health Commission has proactively integrated HCWs into vaccination campaigns through vaccine prescription strategies. This approach aligns with the nation's policy of combining medical treatment with disease prevention [14], positioning vaccine prescriptions as vaccine recommend measures equivalent to medical prescriptions. Shandong, Zhejiang provinces and other places have begun to explore whether clinicians and general practitioners in medical institutions can prescribe vaccines to patients suitable for vaccination [15, 16], and HCWs can provide vaccine prescriptions and provide vaccine recommendations as an innovative measure to integrate vaccination recommendations into the clinical diagnosis and treatment process. It can be recommended as a medical prescription or health education prescription to the appropriate vaccination population [17]. However, the actual implementation and effectiveness remain uncertain, and there is a dearth of research addressing HCWs’ willingness to engage in these interventions.

Despite the critical role of HCWs play in promoting vaccination, the determinants of their willingness to provide vaccine prescription remain insufficiently explored. This study aims to address these gaps by examining the factors that influence HCWs’ willingness in providing vaccine prescription, including workplace characteristics, personal recommendation behaviors, and incentives. This study generates empirical evidence to support the implementation of influenza vaccination prescription strategy and offers valuable insights for global influenza prevention and control efforts. Ultimately, it aligns with the WHO’s Immunization Agenda 2030 (IA2030) [18], which prioritizes the engagement of HCWs to improve vaccine uptake.

## Methods

### Study design

The survey was conducted during the 2024 World Influenza Conference, an event co-hosted by the Chinese Preventive Medicine Association, the Asia–Pacific Alliance for Influenza Control, the Chinese Center for Disease Control and Prevention, and the Chinese Academy of Medical Sciences. The Conference focused on attracting participants including healthcare professionals, public health workers, researchers, and students. Based on data from the 2022 and 2024 conferences, the event had an average of approximately 50,997 total views and 29,897 unique participants, with engagement spanning 6–7 countries. Distinguished from the previous survey questionnaires disseminated by our institution [19, 20], this year’s survey was tailored to the current landscape of influenza vaccination in China, with a particular emphasis on the recommendation practices and willingness of providing vaccine prescription.

### Study population

The sample size was determined by using the formula $\text{n}=\frac{{z}_{1-\alpha }^{2} * p * (1 - p)}{{d}^{2}}$, with a significance level α = 0.05 and a margin of error set at 0.02. Based on previous literature, an estimated influenza vaccination rate of 45% among HCWs was adopted to calculate the minimum sample size required for the study [20], which was 2377. To accommodate potential dropouts, this figure was increased by 10%, resulting in a final minimum sample size of 2614. The survey was designed with flexibility to expand the sample size if financial resources were available. Informed consent was secured from participants at the outset of the survey, with each participant allowed to respond only once. All data collected were anonymized to protect participant privacy. Participants were excluded from the analysis if their responses were from the analysis, e.g. affirming a willingness to receive vaccine while disagreeing with related statements, or vice versa. No response also led to exclusion from the study.

### Variables

The core independent variables in this study included prescription willingness and recommendation behavior. Prescription willingness was defined as HCWs' self-reported intention to prescribe influenza vaccines to eligible patients during the upcoming influenza season, measured via a questionnaire item (willing or unwilling). Recommendation behavior was defined as the actual provision of influenza vaccine recommendation to patients before our study, also assessed through self-reported responses (response options: recommend or did not recommend).

In addition, several control variables included demographic factors (age, gender), professional characteristics (years of work experience, type of workplace, professional title), and vaccination-related factors (history of influenza vaccination, availability of on-site vaccination facilities, and free vaccination policies). The WHO The behavioural and social drivers of vaccination framework (BeSD) framework outline key behavioral and social factors influencing vaccine uptake [25]. Our study categorized the influence factors with the BeSD framework and developed the questionnaire based on its four core domains: motivation, practical issues, social processes, and vaccination (See Table 1). All statistical tests were two-sided, with *P* < 0.05 considered statistically significant.

**Table 1:** Explanatory variables for vaccine prescription and recommendation behaviors

Variable category	Variable name	Description
Demographic Characteristics	Gender, Location	Participant’s gender, work area
	Professional Title, Work Experience	Classification of professional qualifications
	Underlying Medical Condition	HCWs' personal underlying diseases
	Vulnerable population	Patients with chronic underlying diseases, children, students, pregnant women, and the elderly
Incentive factors	Bonus Incentives, Annual/monthly performance evaluations	Role of financial incentives after providing prescriptions
	Learning and training opportunities	Impact of training and educational opportunities after providing prescriptions
	Individual honors, Professional title advancement	Honor and spiritual encouragement after providing prescriptions
	Policy requirements	Established by official regulatory bodies such as national or local Health Commissions
	Hospital-Specific Regulations	Policy mandates published at the institutional level by hospitals, based on directives from the Health Commissions
BeSD-Motivation	Willingness for Next Season	Participant's intention to vaccinate in the upcoming season
BeSD-Pratical issuse	On-Site Vaccination at Workplace	Availability of workplace vaccination facilities
	Free Vaccination Policy	Existence of free vaccination policies at the workplace
	Free policy	Free vaccination policy for participants
	Nearby vaccine clinic	Prescribe the vaccine, allowing patients to receive it at the clinic or nearby vaccination sites
BeSD-Social processes	Recommendation History	Participant's history of recommending vaccines to others
	Objectives: Vulnerable population, Families, Colleagues, Friend	Target population recommended by participants
	Doctor's advice	Include vaccination advice in medical advices
	Health education	Incorporate vaccination into oral health education based on patient circumstances
BeSD-Vaccination	Vaccination History	Participant’s vaccination status for the 2023–2024 influenza season

### Questionnaire development

An anonymous, web-based questionnaire was developed to assess HCWs’ attitudes and behaviors concerning influenza vaccine prescription and recommendation. The questionnaire was designed based on a comprehensive review of relevant literature [21], including studies on vaccine hesitancy, vaccination behavior, and incentive strategies. The development process involved collaboration with three senior public health professionals who provided essential feedback and revisions before the questionnaire’s formal deployment.

The questionnaire was structured into three main sections: (i) Demographic and professional information. This section gathered basic information about the participants, including their workplace type, professional title, and vaccination history, recommendation history; (ii) Key factors influencing willingness of providing vaccine prescription. This section explored key determinants such as institutional policies, availability of on-site vaccination facilities, and previous experiences with recommending vaccines; and (iii) Incentives and strategies to enhance willingness to prescribe vaccines. This section examined various motivators, including financial rewards, opportunities for professional advancement, and training programs. See the supplementary material for further details. (See Supplementary material 1).

Demographic and professional factors are known to influence healthcare workers' attitudes and behaviors. For example, studies have shown that professional role and workplace settings can impact vaccine recommendation practices [22]. Behavioral theories, such as the health belief model and theory of planned behavior, emphasize the role of external factors and past experiences in shaping health-related behaviors [23]. Incentives suggest can significantly encourage healthcare workers’ willingness to engage in specific behaviors [21].

In addition to assessing demographic information, key determinants of vaccine prescription behavior, and incentive strategies, the questionnaire also included items evaluating the effectiveness of digital and direct communication tools in promoting influenza vaccination from HCWs. These tools encompassed public account promotions, SMS reminders, phone call reminders, and technology-driven science education activities. HCWs were asked to indicate which methods they perceived as effective in encouraging vaccine uptake. Participants were asked to rank the importance of different reminder methods on a five-level scale, with Level 1 indicating the highest perceived importance and Level 5 the lowest.

Before formal deployment, the questionnaire underwent pilot testing with a sample of 50 HCWs to evaluate its clarity, relevance, and feasibility. Feedback from the pilot test indicated that the questionnaire was well-structured Specifically, we refined the classification of workplace types and clarified the target populations for vaccine recommendation based on pilot feedback.

### Data collection and processing

Between July 3 and July 10, 2024, a link to the questionnaire for the survey was posted on Listening to the Experts, a reputable learning and communication platform in China that authenticates the identity of its registered users [24]. HCWs who completed the registration on the platform could participate in the responses, not limited to in- person participants. The data collection and processing were carried out by a dedicated team of five members to ensure accuracy and reliability. The data collection phase involved three team members who worked in coordination to systematically gather the required information. They designed a survey aligned with the research objectives, conducted a pilot test to ensure clarity and relevance, distributed the survey questionnaire to participants during the conference via an online platform, and compiled responses into a centralized database for subsequent analysis. The data analysis and verification were carried out by two team members, with each individual's work cross-checked by the other to minimize errors and enhance the consistency of results. Similarly, data processing was handled collaboratively by two members, who performed tasks such as cleaning, coding, and organizing the data for analysis.

### Statistical analysis

Only respondents with complete datasets were included in the final analysis. RStudio (version 2024.04.2 + 764) was used for data analysis. Descriptive statistics, including frequencies, proportions, and means, were used to summarize the sociodemographic characteristics of the HCWs and their influenza vaccination behaviors. Bar charts were created by using the R package “ggplot2” to visually represent key findings.

Bivariate logistic regression models were constructed with the R package “dplyr” to evaluate the associations between HCWs’ willingness to recommend influenza vaccination and variables such as workplace type, professional title, and vaccination history. Based on prior literature and research experience [20], key potential confounders were included in the models as control variables to adjust for their influence on the outcomes of interest.

## Results

### Characteristics of participants

The study encompassed 3140 HCWs distributed across community settings (N = 2009) and hospital settings (N = 1131), revealing significant differences in various sociodemographic characteristics. Females constituted a larger proportion in community settings (73.92%) compared to hospital settings (60.21%), with this difference being statistically significant (*P* < 0.001). Hospital workers were more likely to have higher professional titles, with senior professionals accounting for 1.24% compared to 0.45% in community settings (*P* < 0.001). Experience levels showed significant variation, with hospital workers having fewer than 5 years of experience at a rate of 50.57%, compared to 38.38% among community workers, while community workers had a higher proportion with over 16 years of experience, at 27.38% compared to 15.12% for hospital workers (*P* < 0.001). Additionally, the availability of on-site vaccination at workplace was significantly higher in community settings (87.76%) than in hospitals (82.32%) (*P* < 0.001). (See Table 2).

**Table 2:** Sociodemographic characteristics of study participants

Characteristic	Community (N = 2009)		Hospital (N = 1131)		X^2^	*P* value
	N	%	N	%		
Gender						
Female	1485	73.92	681	60.21	63.522	*P* < 0.001
Male	524	26.08	450	39.79		
Professional title						
Senior	9	0.45	14	1.24	24.229	*P* < 0.001
Sub-Senior	166	8.26	71	6.28		
Middle	703	34.99	473	41.82		
Junior	908	45.2	472	41.73		
None	223	11.1	101	8.93		
Work experience						
≤ 5 years	771	38.38	572	50.57	72.52	*P* < 0.001
6–15 years	688	34.25	388	34.31		
≥ 16 years	550	27.38	171	15.12		
Underlying medical condition						
No	1699	84.57	970	85.76	0.811	0.368
Yes	310	15.43	161	14.24		
On-site vaccination at workplace						
Yes	1763	87.76	931	82.32	17.65	*P* < 0.001
No	246	12.24	200	17.68		

### Willingness to recommendation

**Fig. 1:**
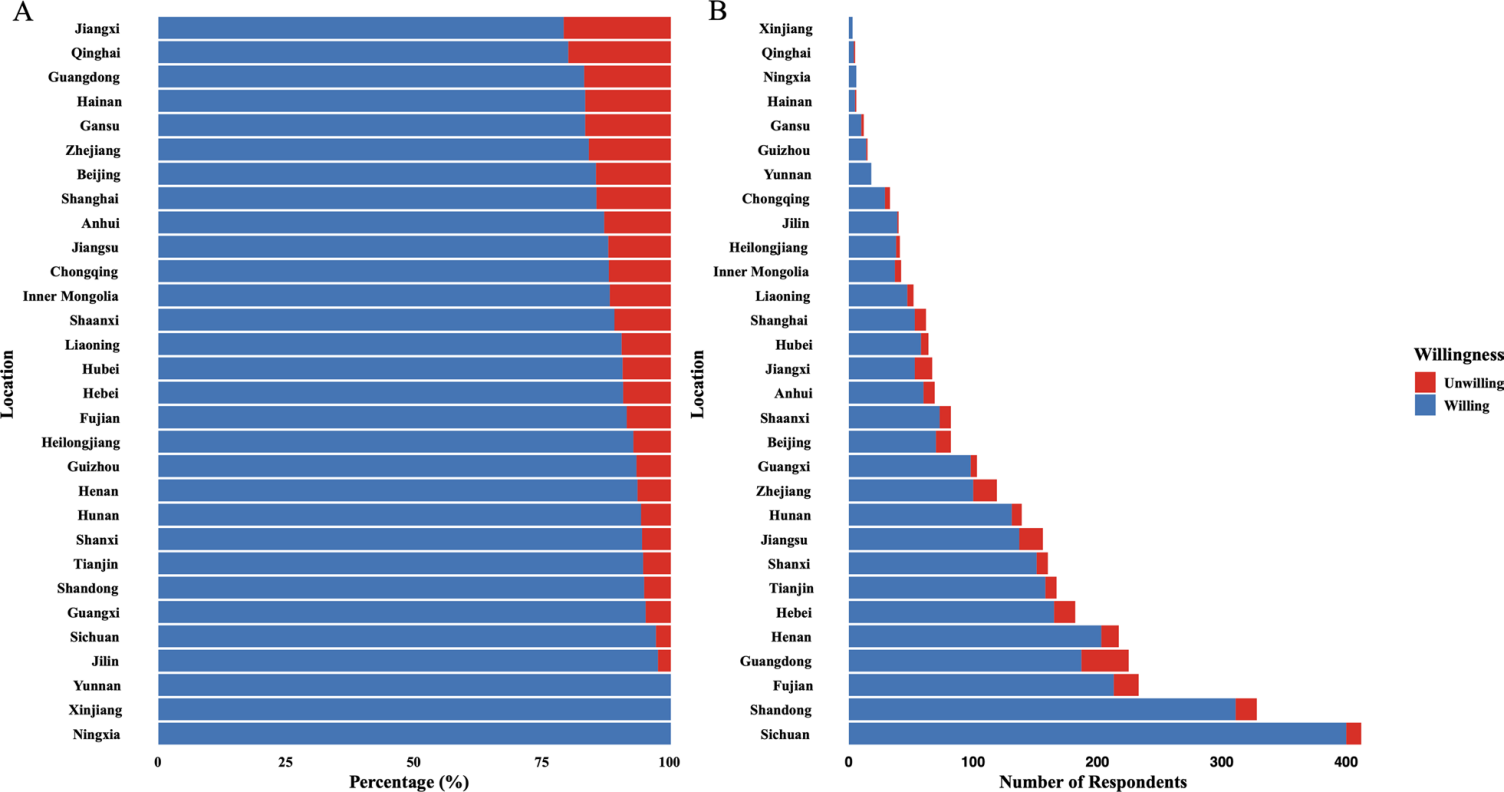
Distribution of attitudes toward providing vaccine prescriptions (**A**) Percentage; (**B**) Number of respondents

In the study, 91.4% (N = 2871) of participants indicated a willingness to provide vaccine recommendation, while 8.6% (N = 269) did not. Geographically, the top three provinces with the highest number of respondents were Sichuan (412, 13.1%), Shandong (328, 10.4%), and Guangdong (225, 7.2%). Respondents from these regions constituted a substantial proportion of the total, providing valuable insights into regional differences in vaccine recommendation willingness. (Fig. 1).

### HCWs'vaccine recommendation practices

Community-based HCWs showed higher recommendation rates than hospital-based HCWs across all groups: vulnerable population (76.21% vs. 72.06%, *P* < 0.05), families (65.16% vs. 56.50%, *P* < 0.05), friends (54.41% vs. 40.41%, *P* < 0.05), and colleagues (48.93% vs. 35.54%, *P* < 0.05). Both community- and hospital-based HCWs emphasized the importance of vaccine prescriptions in guiding individuals to nearby vaccination centers (61.87% vs. 68.79%; *P* < 0.05). Additionally, verbal health education was more commonly prioritized in community health centers (53.71% vs. 43.15%; *P* < 0.05). Another notable recommendation method, doctor’s advice on medical records, was slightly more common in community settings (46.09% vs. 41.20%; *P *< 0.05), highlighting the importance of direct medical guidance in promoting vaccination (See Table 3).

**Table 3:** HCWs’ Recommend behavior of influenza vaccine

Variable		Hospital (N = 1131)		Community (N=2009)		*P* Value
		N	%	N	%	
Recommend objectives						
Vulnerable population	Yes	815	72.06	1531	76.21	0.010
	No	316	27.94	478	23.79	
Families	Yes	639	56.50	1309	65.16	*P* < 0.001
	No	492	43.50	700	34.84	
Colleagues	Yes	402	35.54	983	48.93	*P* < 0.001
	No	729	64.46	1026	51.07	
Friends	Yes	457	40.41	1093	54.41	*P* < 0.001
	No	674	59.59	916	45.59	
Form of recommendation						
Nearby vaccine clinic	Agree	778	68.79	1243	61.87	*P* < 0.001
	Disagree	353	31.21	766	38.13	
Doctor's advice	Agree	466	41.20	926	46.09	0.008
	Disagree	665	58.80	1083	53.91	
Health education	Agree	488	43.15	1079	53.71	*P* < 0.001
	Disagree	643	56.85	930	46.29	

### Factors that influence willingness of providing vaccine prescriptions

As shown in Table 4, the presence of on-site vaccination centers greatly increased willingness of providing vaccine prescriptions (aOR = 8.29, 95% CI: 6.35–10.82, *P* < 0.001), while their absence posed a major barrier. Similarly, awareness of free vaccination policies enhanced willingness of providing vaccine prescriptions (aOR = 1.74, 95% CI: 1.28–2.35, *P* < 0.001), whereas unclear policy access had a negative impact on it (aOR = 10.00, 95% CI: 7.15–13.98, *P* < 0.001). Personal factors also influenced willingness of providing vaccine prescriptions. HCWs with a history of vaccination (aOR = 0.30, 95% CI: 0.23–0.39, *P* < 0.001) or prior recommendation experience (aOR = 99.69, 95% CI: 69.41–143.18, *P* < 0.001) were more likely to provide vaccine prescriptions. Additionally, willingness to receive vaccines for the upcoming vaccination season (aOR = 17.99, 95% CI: 13.45–24.06, *P* < 0.001) and more years of work experience were associated with a higher willingness of providing vaccine prescriptions.

**Table 4:** Factors that influence willingness of providing vaccine prescriptions among HCWs (Ref: Willing to provide prescription)

Characteristic	Unwilling to provide prescription (N = 1119)		Willing to provide prescription (N = 2021)		X^2^	*P* value	OR (95%CI)	*P* value		aOR (95%CI)	*P* value
	N	%	N	%							
Gender											
Female	303	27.08	671	33.20	2.5	0.111	–		–		
Male	816	72.92	1350	66.80							
Work experience											
< 5 years	486	43.43	857	42.40	89.1	P < 0.001	Ref		Ref		
6–15 years	370	33.07	706	34.93			0.70(0.46–1.07)	0.102		0.46(0.34–0.61)	***P*** < 0.001
> 16 years	263	23.50	458	22.66			0.21(0.10–0.44)	***P*** < 0.001		0.13(0.07–0.22)	***P*** < 0.001
Underlying medical condition											
No	962	85.97	1707	84.46	4.9	0.027	Ref				
Yes	157	14.03	314	15.54			1.01(0.57–1.79)	0.973		0.64(0.43–0.95)	0.029
Type of facility											
Community health center	766	68.45	1243	61.5	1.8	0.179	–		–		
Hospital	353	31.55	778	38.5							
Vaccination history (2023–2024)											
Unvaccinated	547	48.88	615	30.43	89.0	***P*** < 0.001	Ref				
Vaccinated	572	51.12	1406	69.57			1.09(0.73–1.65)	0.67		0.30(0.23–0.39)	***P*** < 0.001
Willingness for next season											
Willing	749	66.93	1815	89.81	591.8	***P*** < 0.001	Ref				
Hesitant	370	33.07	206	10.19			6.98(4.69–10.39)	***P***		17.99(13.45–24.06)	***P*** < 0.001
On-site vaccination at workplace											
Yes	866	77.39	1828	90.45	467.6	***P*** < 0.001	Ref				
No	253	22.61	193	9.55			2.73(1.79–4.16)	***P*** < 0.001		8.29(6.35–10.82)	***P*** < 0.001
Free policy											
Yes	428	38.25	1278	63.24	249.3	***P*** < 0.001	Ref				
No	511	45.67	654	32.36			1.41(0.90–2.21)	0.137		1.74(1.28–2.35)	***P*** < 0.001
Unclear	180	16.09	89	4.40			1.49(0.85–2.60)	0.165		10.00(7.15–13.98)	***P*** < 0.001
Recommendation history											
Yes	912	81.50	1985	98.22	1497.8	***P*** < 0.001	Ref				
None	207	18.50	36	1.78			36.16(23.66–55.27)	***P*** < 0.001		99.69(69.41–143.18)	***P*** < 0.001

### Incentives that promote willingness of providing vaccine prescriptions

As shown in Table 5, financial incentives such as bonus rewards were particularly effective in increasing willingness to provide vaccine prescriptions (aOR = 1.84, 95% CI 1.40–2.43, *P* < 0.001). Career-related motivators, including inclusion in annual/monthly performance evaluations (aOR = 1.60, 95% CI 1.20–2.13, *P* = 0.001) and recognition through individual honors (aOR = 1.71, 95% CI: 1.25–2.35, *P* = 0.001) also had a significant impact. Learning and training opportunities also played a crucial role, with HCWs who had access to these resources showing higher willingness (aOR = 2.33, 95% CI: 1.74–3.12, *P* < 0.001). Institutional support, such as the implementation of hospital-specific regulations, further encouraged willingness of providing vaccine prescriptions (aOR = 1.48, 95% CI: 1.15–1.91, *P* = 0.002).

**Table 5:** Incentives factors for providing vaccine prescriptions among HCWs (Ref: Unwilling to provide prescription)

Characteristic	Unwilling to Provide prescription (N = 1119)		Willing to Provide prescription (N = 2021)		X^2^	*P* value	OR(95%CI)	*P* value	aOR(95%CI)	*P* value
	N	%	N	%						
Bonus incentives										
Disagree	805	71.94	1052	52.05	19.35	***P*** < 0.001	Ref		Ref	
Agree	314	28.06	969	47.95			1.58(1.16–2.14)	0.003	1.84(1.40–2.43)	***P*** < 0.001
Annual/monthly performance evaluations										
Disagree	859	76.76	1234	61.06	10.27	0.001	Ref		Ref	
Agree	260	23.24	787	38.94			1.19(0.87–1.64)	0.275	1.60(1.20–2.13)	0.001
Individual honors										
Disagree	901	80.52	1367	67.64	11.39	0.001	Ref		Ref	
Agree	218	19.48	654	32.36			1.13(0.79–1.60)	0.503	1.71(1.25–2.35)	0.001
Professional title advancement										
Disagree	966	86.33	1489	73.68	0.07	0.795	–		–	
Agree	153	13.67	532	26.32						
Learning and training opportunities										
Disagree	723	64.61	1160	57.40	33.82	***P*** < 0.001	Ref		Ref	
Agree	396	35.39	861	42.60			2.45(1.81–3.32)	***P*** < 0.001	2.33(1.74–3.12)	***P*** < 0.001
Policy requirements										
Disagree	548	48.97	1082	53.54	3.98	0.046	Ref		Ref	
Agree	571	51.03	939	46.46			0.88(0.67–1.16)	0.37	0.78(0.60–1.00)	0.046
Hospital-specific regulations										
Disagree	722	64.52	1330	65.81	9.33	0.002	Ref		Ref	
Agree	397	35.48	691	34.19			0.66(0.50–0.86)	0.002	1.48(1.15–1.91)	0.002

### Effectiveness of digital and direct communication tools in vaccine promotion

**Fig. 2:**
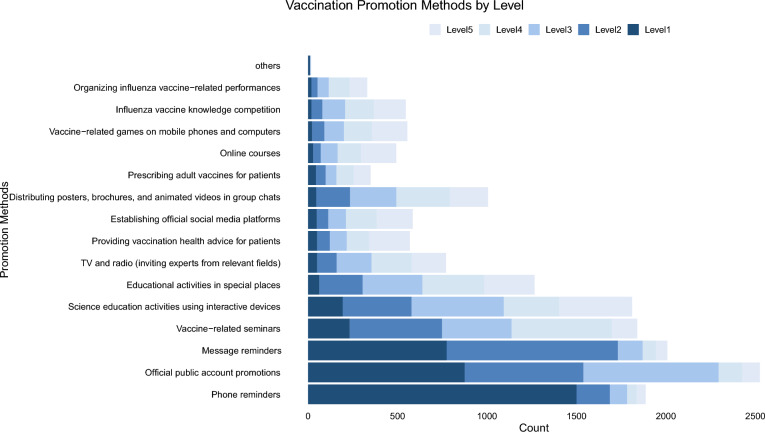
Different forms of reminders for the public to get vaccinated against influenza

As shown in Fig. 2, promotions through official public accounts emerged as the most effective tool, receiving responses from 63.4% of HCWs across all levels, underscoring the crucial role of digital platforms in vaccine education. Phone reminders were particularly notable, with 1501 responses at Level 1, representing 37.7% of HCWs, highlighting the power of direct, personalized communication in encouraging vaccination. SMS reminders also played a significant role, with 50.4% of respondents recognizing their effectiveness. Science education activities using interactive devices were valued by 45.5% of respondents, highlighting the impact of technology-driven approaches.

## Discussion

Our study differentiates between recommendation behavior and willingness of providing vaccine prescriptions among healthcare workers (HCWs), providing contribution to the understanding of gaps in vaccine promotion efforts. Previous studies have also observed a similar gap between willingness and behavior. For instance, despite 96% of HCWs indicating willingness to update their vaccination status, only 24% reported being fully vaccinated [26]. This discrepancy can be attributed to several factors, including lack of infrastructure, time constraints, and competing healthcare priorities. Previous studies have also shown that disparities in healthcare capacity significantly contribute to this issue, with regions having limited healthcare resources more likely to report lower vaccination rates [27].

Our study provides further insights by identifying different determinants for willingness versus actual behavior. Workplace characteristics, particularly the availability of on-site vaccination services, significantly influenced willingness of providing vaccine prescriptions. HCWs working in hospitals, which typically have better infrastructure and institutional support, were more likely to provide vaccine prescriptions compared to those in community health centers. These findings suggest that improving workplace infrastructure, such as establishing on-site vaccination centers—is critical for reduce the gap between willingness and behavior.

Personal factors also influence HCWs' willingness to prescribe vaccines. It is also important to emphasis the relationship between HCWs’ personal vaccination behavior and their willingness to prescribe vaccines. Studies have consistently shown that HCWs who receive vaccinations themselves are more likely to recommend or prescribe vaccines to patients [21]. Studies consistently show that HCWs with prior experience in vaccination are more confident and proactive in recommending vaccines [28]. This aligns with our findings, HCWs who have had previous vaccination experience or have recommended vaccines in the past were more likely to provide vaccine prescriptions. Furthermore, younger HCWs or those with fewer years of experience demonstrated higher rates of vaccine recommendation, which may reflect greater exposure to updated healthcare training and guidelines [29]. Supporting this interpretation, a study conducted in China found that newly trained HCWs were more likely to adopt evidence-based practices compared to their more experienced counterparts, whereas longer work experience was associated with poorer personal protective behaviors [30]. However, in our study, HCWs with more work experience tended to provide vaccine prescriptions, likely due to their exposure to professional training and guidelines. This highlights the importance of continuous professional development in promoting vaccine prescription awareness across healthcare settings.

Our study found that there were regional policy differences in providing vaccine prescriptions willingness across 30 provinces in China, with provinces like Sichuan, Shandong, and Guangdong showing higher participation rates. This is consistent with previous study that reported regional policies and local healthcare infrastructure significantly influence HCWs’ awareness and willingness to recommend influenza vaccines [31]. Shandong’s “Adult Vaccine Prescription Pilot Work Plan” and its related training programs have impacted HCWs’ willingness to recommend vaccines [32], highlighting the direct influence of policy initiatives on the behavior of providing vaccine prescriptions [16, 33]. Guangdong and Sichuan Centers for Disease Control and Prevention have successively piloted vaccine prescriptions for general practitioners to prescribe vaccines to patients who are suitable for vaccination, which is used to recommend or guide patients to receive specific vaccines [34, 35]. Several studies suggest that strategies to improve vaccine uptake among HCWs include enforcing local authority recommendations, implementing practice guidelines, and providing convenient vaccination facilities [36]. The success of these policies suggests that strengthening local health policies, offering specialized training, and incentives for HCWs, and ensuring infrastructure support in regions like Sichuan and Guangdong could be effective strategies for improving vaccine prescription practices. However, long-term studies are needed to evaluate the sustained impact of these policies and whether they result in a significant increase in vaccination coverage.

Vaccine prescription policies differ internationally. In the United States [37], pharmacies and digital health records are key components of vaccine prescription. Pharmacies have become key players in vaccine delivery [38], with nearly 60% increase in flu vaccinations administered by pharmacists [39]. This model offers patients more convenience and accessibility, which has led to higher vaccination rates. In comparison, China's vaccine prescription model relies heavily on community health service centers and doctor recommendations [40]. A previous study found that this model has contributed to a strong primary healthcare infrastructure, but it still faces challenges in achieving high vaccine uptake, particularly in rural areas. While China’s approach has strengths, including its extensive primary care network, further studies are needed to assess whether digital health solutions and pharmacy-based vaccinations could enhance the current system and increase vaccine access, particularly in underserved areas.

However, this study has several limitations. First, there may be selection and reporting biases related to the study population and data collection methods. Participants were recruited during the 2024 World Influenza Conference, and although both in-person and online attendees were eligible, HCWs who participated may have been more professionally active or motivated than the general healthcare workforce. In addition, vaccine recommendation behavior was self-reported, which may introduce recall bias or social desirability bias, potentially leading to an overestimation of positive behaviors. These factors may limit the generalizability and accuracy of the findings. Second, the study did not account for contextual factors specific to healthcare institutions, such as organizational culture, workload, or resource availability, which may influence HCWs’ willingness and behavior regarding vaccine prescription. Future studies incorporating qualitative methods, such as interviews or focus groups, could provide deeper insights into these contextual differences. Third, as vaccine prescription practices are still in the early stages of implementation in China, there may be inconsistencies in participants’ interpretation of what constitutes a “vaccine prescription.” Standardized definitions and intervention evaluations are needed to assess the effectiveness and impact of vaccine prescription programs on vaccination uptake.

## Conclusions

This study highlights the need for incentive strategies to enhance healthcare workers’ vaccine prescription practices and improve vaccination rates. Bridging the gap between willingness and practice requires a multifaceted approach involving policy interventions, improved healthcare facilities, and ongoing training. Further research is needed to evaluate the long-term impact of vaccine prescription practices. By strengthening local healthcare systems and integrating effective digital health tools, China can make significant strides in improving vaccine uptake and contribute to global efforts in combating vaccine-preventable diseases.

## Supplementary Information

Additional file 1.

## Data Availability

The data that support the findings of this study are available from the corresponding author upon reasonable request.

## Abbreviations

HCWs: Healthcare workers
CI: Confidence interval
OR: Odds ratio
CHWs: Community health workers
SDG: Sustainable development goals
