# Supplementary material for: Healthcare workers’ attitudes toward influenza vaccine prescriptions in China
Source: Glob Health Res Policy. 2025 Aug 4;10:34. doi: 10.1186/s41256-025-00430-0 (PMC12323280; doi:10.1186/s41256-025-00430-0)
Supplement: Supplementary file 1 — Additional file 1. [file 41256_2025_430_MOESM1_ESM.docx]

Supplementary material

**Survey on Influenza Vaccine Recommendation Attitudes among Public Health Workers**

Dear colleagues,

Greetings and warmest thanks for joining us at the 2024 World Influenza Conference. Public health workers play an indispensable role in disseminating vaccine-related knowledge and providing vaccination services. Health education stands as an important measure to improve the health literacy of the broader community. Therefore, to understand current public health workers' attitudes towards recommending vaccines, and their engagement in such activities, this survey is jointly conducted by the Chinese Preventive Medicine Association, the School of Population Medicine and Public Health at Peking Union Medical College, and the magazine *Three Generations*, aiming to provide a basis for the advocacy efforts for public influenza vaccination.

Conducted during the 2024 World Influenza Conference, this survey will strictly uphold principles of personal privacy protection and will not include any judgment regarding individual behaviors or choices Your participation and active cooperation are invaluable to our efforts to improve influenza prevention and vaccine application in China. We greatly appreciate your contribution!

**1.Are you a healthcare worker?**
☐ Yes
☐ No (Terminate the survey)

**Section 1: Basic Information**

**2.Your gender:**
☐ Male
☐ Female

**3.Province where your workplace is located:**
☐ [Dropdown options]

**4.Your professional title:**
☐ None
☐ Junior
☐ Middle
☐ Sub-Senior
☐ Senior

**5.Years of experience in healthcare:**
☐ [Dropdown numeric options]

**6.Do you have any of the following chronic diseases? (Select all that apply):**
☐ None (Mutually exclusive)
☐ Hypertension
☐ Diabetes
☐ Cardiovascular disease
☐ Cerebrovascular disease
☐ Respiratory diseases (e.g., asthma, COPD)
☐ Chronic kidney disease
☐ Chronic liver disease
☐ Immunodeficiency
☐ Cancer
☐ Other __________

**7.Type of healthcare facility where you work:**
☐ Hospital (tertiary or secondary)
☐ Township or community health center
☐ Other (e.g., emergency center, testing center, publishing house, health administration, etc.) (please specify __________)

**Section 2: Personal Influenza Vaccination Behavior**

**8.Between September 2023 and April 2024, did you experience respiratory infection symptoms?**
☐ Severe symptoms (required medical attention)
☐ Mild symptoms
☐ No symptoms

**9.Between September 2023 and April 2024, did you receive an influenza vaccine?**
☐ Yes
☐ No

**10.Are you willing to receive the influenza vaccine for the upcoming season (2024–2025)?**
☐ Yes
☐ No
☐ Undecided

**11.Does your workplace have a vaccine clinic?**
☐ Yes
☐ No
☐ Unsure

**12.Does your workplace offer free influenza vaccination for employees?**
☐ Yes
☐ No
☐ Unsure

**Section 3: Attitudes Toward Recommending Influenza Vaccination**

**13.Have you previously recommended influenza vaccines to others?**
☐ Yes, to key populations (e.g., healthcare workers, patients with chronic diseases, children)
☐ Yes, to family members
☐ Yes, to colleagues
☐ Yes, to friends
☐ No

**14.When you previously recommended influenza vaccination, did it change the recipient's attitude towards vaccination?**
☐ Yes, they successfully received the vaccine
☐ Yes, they expressed willingness but were unsure if they received it
☐ No, they decided against receiving it
☐ No, they remained hesitant
☐ Unsure, I did not follow up

**15.Are you willing to recommend influenza vaccination to others this year?**
☐ Yes, to key populations (e.g., healthcare workers, patients with chronic diseases, children)
☐ Yes, to those around me (e.g., family, colleagues, friends)
☐ No (Mutually exclusive option)
☐ Undecided (Mutually exclusive option)

**16.If you have prescribing authority, which form of recommendation do you prefer?**

|  | **Agree** | **Disagree** |
| --- | --- | --- |
| Prescribe the vaccine, allowing patients to receive it at the clinic or nearby vaccination sites | **·** | **·** |
| Include vaccination advice in medical orders | **·** | **·** |
| Incorporate vaccination into oral health education based on patient circumstances | **·** | **·** |

**17.What factors would motivate you to recommend influenza vaccination to others?**

|  | **Agree** | **Disagree** |
| --- | --- | --- |
| Linking each dose administered to performance bonuses | **·** | **·** |
| Including it in annual or monthly performance evaluations | **·** | **·** |
| Tying it to personal honors or awards | **·** | **·** |
| Making it a criterion for professional title promotions | **·** | **·** |
| Providing educational or cultural incentives (e.g., training opportunities) | **·** | **·** |
| Government-issued policies | **·** | **·** |
| Institutional regulations | **·** | **·** |

**18.How effective do you think recommending influenza vaccination is in improving vaccine coverage?**
☐ Very effective
☐ Moderately effective
☐ Not significantly effective

**19.Which methods of recommendation do you think are most effective? Please select and rank the top 5:**
☐ Phone call reminders
☐ Text message reminders
☐ Official public account promotions
☐ Vaccine-related lectures
☐ Public education activities with interactive devices (e.g., touch screens, VR)
☐ Distribution of posters, brochures, or animation videos in groups
☐ Education at special locations (e.g., schools, nursing homes)
☐ Television and radio promotions featuring experts
☐ Vaccine knowledge competitions
☐ Interactive online games related to vaccine knowledge
☐ Dramas, skits, or talk shows about influenza vaccination
☐ Online courses targeting key populations (e.g., healthcare workers, older adults)
☐ Official social media platforms for vaccine knowledge dissemination (e.g., Weibo, Xiaohongshu)
☐ Adult vaccine prescriptions
☐ Establishment of adult preventive clinics
☐ Other __________
